# Future teachers and the self-perception of vocal symptoms and knowledge about vocal health and hygiene

**DOI:** 10.1590/2317-1782/20232022160en

**Published:** 2023-10-09

**Authors:** Inaiê Caroline Brugnolo Rosa, Ana Paula Dassie-Leite, Eliane Cristina Pereira, Perla do Nascimento Martins

**Affiliations:** 1 Universidade Estadual do Centro-Oeste - UNICENTRO - Irati (PR), Brasil.

**Keywords:** Voice, Teachers, Voice Disorders, Health Promotion, Quality of Life

## Abstract

**Purpose:**

To analyze responses of future teachers regarding the self-perception of vocal symptoms and knowledge about vocal health and vocal hygiene, relating them to sociodemographic and occupational variables and previous knowledge about voice.

**Methods:**

Observational, analytical and cross-sectional study. A total of 264 undergraduate students participated and the Vocal Health and Hygiene Questionnaire (QSHV), Vocal Symptoms Scale (ESV) and a questionnaire with sociodemographic, occupational and vocal questions were applied. Data were statistically analyzed considering a 5% significance level.

**Results:**

QSHV of future teachers had a total mean score of 21.89, suggestive of lack of knowledge about vocal health and hygiene. The results of the ESV are above the instrument's cutoff score, in each of the three domains and in the total score, there was greater symptomatology in first-year students when compared to other students, in the emotional, limitation and total domains. There was a difference when comparing the QSHV with the year of graduation (p=0.001), in which third and fourth year students obtained higher scores. A positive correlation was observed between the total QSHV score and the age variable (p=0.019).

**Conclusion:**

Future teachers present important vocal symptomatology, compatible with dysphonic individuals, and have insufficient knowledge about vocal health and hygiene. Knowledge is even lower among students in the early undergraduate years and at a younger age. Older future teachers demonstrate more knowledge about vocal health and hygiene. It is necessary to propose preventive actions with this population, even during the graduation period, aiming to reduce the risks of developing vocal problems in the medium or long term during teaching.

## INTRODUCTION

Teachers are voice professionals with high vocal demand and they start their teaching activities even before they finish their academic training, during their undergraduate courses. In their work activities, the voice is one of the main work resources, and its quality is of great importance for both professional performance and the teaching-learning process^(1)^.

Compared to other voice professionals, teachers stand out for being at high risk for developing vocal alterations^(2)^. More than half of the teachers have already had or have some vocal alteration^(3)^, and the main symptoms reported by them are vocal fatigue, vocal effort, tiredness when speaking, burning, hoarseness, dry throat, aphonia, hawking, and dry cough^(4)^.

Studies show that Brazilian teachers have a higher perception of vocal symptoms^(5)^. This condition may be related to factors such as high vocal demand, environmental and occupational factors, individual predisposition, difficulty in applying knowledge about vocal health in daily activities, and lack of access to information during their training period^(6)^.

There are few studies in the literature about the voice of future teachers, especially studies related to the perception of vocal symptoms^(6)^ and knowledge of vocal health^(7)^. It is known, however, that during the graduation period, future teachers rarely receive guidance on vocal health, nor do they have activities focused on this issue^(8)^.

A study conducted with future teachers shows that undergraduate students of pedagogy courses have a higher incidence of vocal symptoms when compared to students of other undergraduate courses^(9)^. Another study refers to the existence of hoarse voice in final year undergraduate students of mathematics, literature, and pedagogy, especially among those who already perform some kind of teaching activity, and points out that less than 30% of students reported having habits of voice care^(10)^. Literature shows that by applying the Voice Symptom Scale (VoiSS) it is possible to find higher scores, above the cutoff scores, in subjects with vocal symptoms compared to subjects without vocal symptoms^(11)^. Thus, it is possible to observe that subjects with higher scores show a higher probability of developing dysphonia, thus demonstrating the importance of prevention or health promotion actions, or both for this group.

Regarding vocal health and hygiene procedures, literature points out that teachers demonstrate to have only basic knowledge about habits considered beneficial or that can cause damage to vocal health^(12)^. In the case of future teachers, the most reported beneficial habit was drinking water, ranked as the greatest voice care agent^(13)^. It is worth noting that literature shows that future teachers have insufficient vocal knowledge^(8,13)^, especially to cope with work-related adversities and the occurrence of vocal disorders.

In this context, to investigate the individual's perception of his or her own voice, self-assessment instruments have been widely developed and used^(14,15)^. Among them, the instrument considered most robust for self-assessment of vocal symptoms is the VoiSS^(16,17)^. This scale evaluates three specific domains, related to limitations and physical and emotional aspects, and has been used to investigate the symptomatology and its relation with possible vocal disorders in voice professionals, such as education professionals^(18)^. Regarding knowledge about issues related to vocal health and hygiene, the Vocal Health and Hygiene Questionnaire (VHHQ)^(19)^ has been used for presenting cutoff values that separate people according to the knowledge they have about the subject.

Despite research with prospective teachers and standardized instruments for evaluation, there is still a significant gap in understanding the vocal profile and needs of this population. Research with future teachers in general is directly linked to actions with a bias towards the prevention of dysphonia^(20,21)^, and these studies show that dysphonia is common in the first years of graduation^(20)^ and that actions on voice have a positive effect on this population^(20,21)^. This condition demonstrates the importance of conducting studies to better understand the particularities of this population so that preventive actions can be developed. The intention is to add more information on the subject, especially in the Brazilian literature, considering that, in the category of future teachers, the presence or absence of vocal symptoms and the knowledge of these individuals about vocal health and hygiene are still little studied in the area of Voice. Thus, it is necessary to have more knowledge about this population so that we can think of primary actions aimed at adding knowledge about health and vocal hygiene and, consequently, preventing vocal problems in the medium or long term, or both, during the teaching profession.

Therefore, the aim of this research is to analyze the answers of future teachers regarding the perception of vocal symptoms and knowledge about vocal health and vocal hygiene, relating them to sociodemographic, occupational variables and previous knowledge about voice.

## METHODS

### Study design

This is an observational, analytical, cross-sectional study.

### Ethical Aspects

This research was approved by the Ethics Committee for Human Research, under number 3.282.389.

### Sample

Students enrolled in undergraduate courses at a Brazilian public university were invited to participate in this research, and all participants signed the Informed Consent Form. Dates and locations were previously agreed upon between researcher and participants, and the sample was composed by convenience.

As inclusion and exclusion criteria, the information was verified through questions asked to the participants and were considered: 

Inclusion: being an undergraduate student, regularly enrolled in a degree course. 

Exclusion: having a history of head and neck surgery, neurological problems with an impact on voice and communication; diagnosis of laryngeal or vocal alterations; performing phono-audiological monitoring for rehabilitation or vocal improvement; being a smoker.

### Procedures

The procedures involved in data collection were the responses to the protocols:

- VoiSS - Voice Symptom Scale^(16,17)^: composed of 30 questions that assess the following domains linked to vocal symptoms and impact on life: limitation, which assesses voice-related restrictions; physical, which assesses biological issues; and emotional, which assesses the volunteer's relationship with his voice and the emotional impact it brings. The limitation domain has a maximum score of 60 points, the physical domain has 32 points, and the emotional domain has 28 points. The maximum VoiSS score is 120 points, and the higher the score, the higher the vocal symptomatology. The respective cutoff scores, which separate vocally healthy subjects and people at risk for developing dysphonia, are 11.5 for the limitation domain, 6.5 for the physical domain, 1.5 for the emotional domain, and 16.0 for the total VoiSS score^(17)^.
- VHHQ - Vocal Health and Hygiene Questionnaire^(18)^: composed of 31 questions to measure knowledge about vocal health and hygiene, each with three response options: positive, negative or neutral, considering the items as beneficial, neutral or harmful to the voice. The total score is calculated by simple summation, one point for each correct answer, and the instrument's cutoff value is 23 points, with scores below this value indicating insufficient knowledge and consequent risk for developing a vocal disorder^(18)^.
- Questionnaire on socio-demographic data: the questions asked were pertinent to general socio-demographic issues such as gender, age, year of graduation, occupational activity/work parallel to the university (yes or no), activity outside the university with great vocal demand (work or other activities such as singing in church, participation in singing groups, theater, among others), and whether the participants had received previous guidance on voice use.

### Data analysis

For the data analysis, the results obtained were tabulated in an EXCEL spreadsheet and the analyses were performed using the software *Statistica* for Windows, version 10.0, *StatSoft Inc*. Descriptive analysis of the students' scores on the VHHQ was performed (measures of central tendency and dispersion). To verify the normality of the continuous variables, the Shapiro-Wilk test was applied, and asymmetrical distribution was found for all of them. Therefore, the non-parametric Mann-Whitney test was used for comparison between the scores of the VHHQ or VoiSS instruments and the variables gender, work, other activities with voice use, and voice use orientation. The comparison between the scores of these instruments and the variable graduation year was performed using the Kruskal-Wallis non-parametric ANOVA test (more than two groups), with Tukey HSD post-hoc analysis. Spearman's Test was used for the correlation between the scores of the VoiSS and the VHHQ. In all statistical tests a 5% significance level was adopted (p<0.05).

## RESULTS

A total of 264 students enrolled in six undergraduate courses at a Brazilian university participated in the present study. It is worth mentioning that the university campus involved in the study has seven undergraduate programs, with a total of 920 total openings, considering all undergraduate years. However, due to the dropouts and the low demand that has increased in recent years, especially in the undergraduate courses, the number of students is well below this total. Therefore, it is believed that 264 participants is a significant number given the context. However, it is important to mention the lack of prior sample size calculation as a limitation; this would determine the exact number of participants needed to generalize the results.

The group was composed of students from the first to the final year of undergraduate programs: history, geography, letters-Spanish, letters-English, pedagogy, mathematics, and physical education. Data were collected from 71 first-year students (26.9%), 96 second-year students (36.4%), 48 third-year students (18.1%), and 49 fourth-year students (18.6%).

The group was composed of 167 women, 96 men, and 1 participant who did not report gender, with a mean age of 21.86 years, a minimum of 18 years, and a maximum of 47 years. Only 38 students reported having received previous guidance on voice use (14.4%).

Table 1 shows the descriptive analysis of the results obtained in the VoiSS and VHHQ protocols. The VoiSS results are above the cutoff score of the instrument, in each of the three domains and in the total score. The VHHQ showed in this study a mean score value of 21.89, below the instrument's cutoff score.

**Table 1:** Descriptive analysis of the scores obtained in the Voice Symptoms Scale (VoiSS) and in the Vocal Health and Hygiene Questionnaire (VHHQ), considering the total group of future teachers

**Voice Symptoms Scale (VoiSS)**	**n**	**Average**	**Median**	**Min.**	**Max.**	**SD**
**Physical**	264	7.29	7.00	0.00	21.00	4.49
**Emotional**	264	3.62	1.00	0.00	26.00	5.16
**Limitation**	264	17.66	16.00	0.00	47.00	8.68
**Total**	264	28.56	25.00	0.00	76.00	15.21
**Vocal Health and Hygiene Questionnaire (VHHQ)**	**n**	**Average**	**Median**	**Min.**	**Max.**	SD
**Total**	264	21.89	24.00	0.00	31.00	6.83

Caption: n = number of participants; Min. = minimum; Max. = maximum; SD = standard deviation

Table 2 shows the comparison between the results of the VoiSS and the variables gender and previous guidance on voice use, making it possible to analyze each domain separately. According to the data obtained, no statistically significant differences were observed for this analysis.

**Table 2:** Comparison of the mean VoiSS score between gender, work, activities outside the university with high vocal demand (occupational or not) and previous guidance on voice use

VoiSS	Women (n: 167)					Men (n: 97)					p
	Average	Median	Min.	Max.	SD	Average	Median	Min.	Max.	SD	
Physical	7.46	7	0	21	4.38	7.04	6	0	21	4.68	0.343
Emotional	3.65	1	0	26	5.44	3.58	2	0	25	4.69	0.517
Limitation	18.2	16	5	47	8.78	16.8	16	0	37	8.5	0.362
Total	29.3	26	6	76	15.55	27.41	25	1	66	14.62	0.458
VoiSS	Previous Orientations - No (n: 226)					Previous Orientations - Yes (n: 38)					p
	Average	Median	Min.	Max.	SD	Average	Median	Min.	Max.	SD	
Physical	7.14	7	0	21	4.48	8.16	8	0	18	4.51	0.167
Emotional	3.45	1	0	26	5.06	4.66	2	0	21	5.69	0.069
Limitation	17.54	16	0	47	8.64	18.34	16.5	6	39	9.05	0.706
Total	28.12	25	1	76	15	31.16	27.5	9	72	16.36	0.34
VoiSS	Works - No (n: 98)					Works - Yes (n: 166)					p
	Average	Median	Min.	Max.	SD	Average	Median	Min.	Max.	SD	
Physical	7.24	6	0	21	4.80	7.31	7	0	21	4.31	0.905
Emotional	3.91	1	0	24	5.48	3.45	1	0	26	4.97	0.489
Limitation	17.61	16	0	41	9.03	17.68	16	1	47	8.50	0.951
Total	28.45	26.5	3	72	15.96	28.45	25	1	76	14.79	0.877
VoiSS	External activities with high vocal demand - No (n: 228)					External activities with high vocal demand - Yes (n: 36)					p
	Average	Median	Min.	Max.	SD	Average	Median	Min.	Max.	SD	
Physical	7.18	7	0	21	4.43	7.94	7.5	0	18	4.85	0.346
Emotional	3.68	1	0	26	5.32	3.28	2	0	16	4.08	0.668
Limitation	17.91	16	0	47	8.94	16.03	15	6	39	6.77	0.227
Total	28.76	26.00	1.00	76.00	15.54	27.25	23.00	8.00	65.00	13.03	0.580

Mann-Whitnney test

Caption: n = number of participants; Min = minimum; Max = maximum; SD = standard deviation; p = probability of result according to the significance level

Below are the results of the comparison between the VoiSS and the undergraduate year variable. More symptomatology is observed in first-year students when compared to students in other years in the emotional, limitation, and total domains (Table 3).

**Table 3:** Comparison between the variable graduation year and mean score on the VoiSS Symptoms Scale (VoiSS), considering the group of future teachers

**Graduation Year**	**VoiSS Physical**						**post-hoc**	**VoiSS Emotional**						**post-hoc**
	**Average**	**Median**	**Min.**	**Max.**	**SD**	**p**		**Average**	**Median**	**Min.**	**Max.**	**SD**	**p**	
First (n:71)	8.06	8.00	0.00	21.00	4.87	0.224		5.49	3.00	0.00	24.00	6.34	**0.005**	1>2=3
Second (n:96)	7.28	7.00	0.00	17.00	4.53			2.86	1.00	0.00	21.00	4.15		
Third (n:48)	5.88	5.00	1.00	15.00	3.20			2.04	1.00	0.00	25.00	4.07		
Fourth (n:49)	7.57	7.00	0.00	18.00	4.71			3.94	2.00	0.00	26.00	5.30		
**Graduation Year**	**VoiSS Limitation**							**VoiSS Total**						
	**Average**	**Median**	**Min.**	**Max.**	**SD**	**p**		**Average**	**Median**	**Min.**	**Max.**	**SD**	**p**	
First (n:71)	20.37	19.00	1.00	47.00	9.43	**0**	1>3=2=4	33.89	32.00	1.00	76.00	17.56	**0**	1>3=2=4
Second (n:96)	17.46	15.50	4.00	40.00	8.11			27.60	25.00	6.00	72.00	13.18		
Third (n:48)	14.08	14.50	0.00	32.00	7.35			22.00	19.50	3.00	55.00	11.76		
Fourth (n:49)	17.61	17.00	4.00	38.00	8.77			29.12	23.00	6.00	75.00	15.86		

Kruskal Wallis ANOVA and Tukey HSD Test

Caption: n = number of participants; Min = minimum; Max = maximum; SD = standard deviation

Regarding the comparison between the Total VHHQ score and the analyzed variables, Table 4 shows a statistically significant result when comparing the variable graduation year (p=0.001), in which students from the last years obtained higher scores. Other variables did not present significant results.

**Table 4:** Comparison between mean score of the Vocal Health and Hygiene Questionnaire (VHHQ) and variables gender, graduation year, previous guidance on voice use, work and activities outside the university with high vocal demand

**Variable**	**VHHQ - Total Score**							**P**	**post-hoc**
	**Group**	**n**	**Average**	**Median**	**Min.**	**Max.**	**SD**		
Gender*	Fem	167	21.57	24	0	31	7.23	0.585	
	Male	96	22.56	24	2	31	5.97		
Graduation Year**	1	71	19.79	22	0	30	7.61	**0.001**	4=3>1=2
	2	96	20.97	22.5	2	30	6.88		
	3	48	24.48	25.5	8	31	5.23		
	4	49	24.2	25	10	31	5.47		
Prior guidance on voice use***	No	226	21.83	24	0	31	6.83	0.633	
	Yes	38	22.24	24.5	4	30	6.89		
Works***	No	98	21.28	23	0	30	7.14	0.269	
	Yes	166	22.24	24	2	31	6.63		
Activities outside the university with high vocal demand***	No	228	21.72	24	0	31	6.87	0.319	
	Yes	36	22.94	25	2	30	6.53		

* Mann Whitney;

** Kruskal Wallis ANOVA and Tukey HSD;

*** u test

Caption: n = number of participants; Min = minimum; Max = maximum; SD = standard deviation

In the findings regarding correlation, Table 5 shows a significant positive correlation between the total score of the VHHQ and the variable age.

**Table 5:** Correlation between the value of the total score of the Vocal Health and Hygiene Questionnaire (VHHQ) and the variables age and total score of the Voice Symptom Scale (VoiSS), considering the group of future teachers

**Variable**	**Age**		**VoiSS - Total scores**	
	**r**	**p**	**r**	**p**
**VHHQ - Total scores**	0.144	0.019*	-0.092	0.138

Spearman Correlation

## DISCUSSION

The voice is considered an essential work resource for approximately 25% of the economically active population^(22)^. Literature shows that the presence of vocal symptoms and lack of knowledge about vocal health and hygiene procedures can compromise the performance of voice professionals^(5,6,23)^. In the case of teachers, vocal alterations can impact teaching activities and compromise the teaching-learning process^(13,23)^.

The original VoiSS (Voice Symptom Scale) was developed from research with over 800 patients to be a robust instrument for self-assessment of voice and vocal symptoms^(16)^. The Portuguese version is one of the instruments with the greatest discrimination power between subjects with vocal alterations of different etiologies and healthy voices, and its validated cutoff values allow the use for screening large populations and individuals at vocal risk^(17)^. In the present research, the VoiSS results are above the instrument's cutoff score in each of the three domains and in the total score, indicating high symptomatology and vocal risk in future teachers.

The results of the VoiSS and VHHQ protocols in this study show that future teachers have a significant perception of vocal symptoms and little knowledge about vocal health and hygiene procedures. In the case of future voice professionals, a high mean score obtained in the VoiSS demonstrates a higher possibility of developing vocal disorders according to the vocal demand required by the profession^(24)^.

The existence of vocal symptoms in the population studied corroborates with findings of other studies, which report that teachers often present vocal symptoms, such as hoarseness, pain when speaking, and voice failure at the end of the day^(23)^. These symptoms may be related to factors such as work environments, organization of work activities, as well as lack of access to information on vocal health procedures, or inability to apply vocal knowledge due to work organization or an unhealthy environment^(1,6,10)^.

The presence of symptoms may also be related to the lifestyle of these future teachers, as it is known that university students can present high vocal symptoms associated with intense vocal use, smoking, stress, and digestive problems^(25)^. The aforementioned study points out that aspects related to habits, health, vocal demand, and external environment seem to influence the appearance of vocal symptoms in university students. These aspects may favor the expressive occurrence of vocal symptoms in this population.

Regarding the results of the VHHQ, the findings show that future teachers demonstrate less knowledge about procedures related to vocal care. Other studies conducted with future teachers also report similar results. Participants reported the presence of high vocal symptomatology^(9)^, little voice care^(10)^, and insufficient knowledge about vocal health and hygiene^(13)^. This condition suggests that future teachers need vocal health guidance programs during the undergraduate period. Such actions are important, mainly because this population starts to work professionally still during academic formation through internships, and the voice is their main work resource. Quality of life influences the proper functioning of the body, which also improves performance in occupational activities. In the case of teachers, voice-related quality of life is an important reference to understand the perception that this population has about their reactions to voice alterations and about their vocal health^(24-25)^.

In this context, it is worth noting that there are few discussions about the effect of dysphonia on student learning. However, one investigation showed that the non-dysphonic voice produced a positive impact to the detriment of the negative effects imposed by the teacher's dysphonic voice^(26)^. This is another reason why there is a need to give importance to the vocal health of teachers since their training process, which unfortunately is still little valued. The absence of voice training in teaching undergraduate programs is noticeable, and a study showed that coordinators of various sectors understand the importance of the voice as a teacher's work tool; however, they only highlight the existence of isolated actions in the curriculum^(8)^.

Regarding the comparison between the undergraduate years, this study shows higher symptomatology in first-year students compared to students from other years for the emotional, limitation, and total VoiSS domains, which is compatible with subjects with vocal alterations. No previous studies comparing future teachers in relation to undergraduate years were found. Considering the findings of this study, this difference may be related to changes in the lifestyle of this population due to university admission, as these future professionals undergo a change in their daily routine, they often change their eating patterns and start to develop academic activities that require greater vocal use and better performance of communication skills. Such situations may lead to vocal use with more effort and phonatory tension, besides generating an increase in insecurity and shyness, factors that may influence the perception of symptoms or vocal disadvantage^(25,27)^. For a better understanding of these circumstances, studies to provide evidence are needed, both to understand the specific differences between university years and to assess the voice of future teachers in a multidimensional way in the different years of undergraduate programs.

On knowledge of vocal health and hygiene procedures, fourth and third-year undergraduate students scored higher on the VHHQ. This finding suggests that the longer the time in the undergraduate program, the more knowledge about vocal health and hygiene is acquired. In the case of the research institution, voice education programs are held every year, where information about vocal health and hygiene is provided to all students in the undergraduate programs. Such actions seem to increase the knowledge of students in their last years of undergraduate programs, as they have participated more times in these activities.

Considering the correlation between the total score of the VHHQ and the variable age, the results show that the older the participants, the greater their knowledge about vocal health and hygiene. Although literature points out that subjects of different generations have basically the same knowledge about habits that benefit and harm vocal health^(28)^, this study shows that the older the undergraduate student is, the greater their knowledge of life. So health and vocal knowledge may come from personal experiences.

In this study, there was no correlation between the total score of the VHHQ and VoiSS. A recent study points out that teachers with more knowledge about vocal health observe a greater perception of fatigue and vocal restriction^(5)^. However, that study only investigated the fatigue symptom. It is hypothesized here that knowledge about vocal health is not always operative, that is, knowing a certain habit may not be enough to modify the practice related to it and, consequently, prevent possible vocal symptoms.

The present study contributes to the scientific knowledge of the area since it shows that future teachers have significant vocal symptoms and lack knowledge about vocal health and hygiene. It also shows that the knowledge is lower in students of early years and with lower age. Therefore, strategies to reduce the risks of developing vocal problems in these future voice professionals should be encouraged. Thus, it is worth emphasizing the importance of developing vocal health actions with this group focused on the knowledge of self-care, contributing to avoiding possible vocal problems. It is understood that experiences have an important role in learning about vocal health and hygiene, being a relevant factor not only for the development of healthy vocal habits and behaviors but also for the improvement of teachers' quality of life.

Therefore, it is important to discuss the need to bring Speech Therapy closer to undergraduate programs, either through extension projects, frequent lectures, or discipline in the curriculum, to prevent the occurrence of future voice problems and ensure the effectiveness of their professional activity through proper vocal use.

The variable referring to teaching activities already practiced by future professionals was not explicitly controlled; however, at the university, where the researched data were collected, the practicum for undergraduate programs usually takes place in the third and fourth years, which allows the inference that they are already performing some tasks in the classroom in these years. Even though there were no statistically significant differences in the results comparing students with and without occupational activities and with and without activities outside the university with high vocal demand, it is suggested that the variable referring to vocal use in other spaces and other ways be better investigated in future research, deepening the analysis on possible overloads of voice use experienced by students, which could influence the results regarding vocal symptoms and knowledge about voice health.

## CONCLUSION

The present study shows that it is possible to verify the existence of vocal symptomatology in undergraduate students regarding the physical, emotional, and limitation aspects of the Voice Symptom Scale, VoiSS. Results show that future teachers have little knowledge about vocal health and hygiene. The knowledge is even lower in students in the first years of undergraduate programs and with lower age, being noticeable that the higher the age of the participants, the greater the knowledge about vocal health and hygiene. Knowledge about vocal health has no direct relationship with vocal symptoms presented by students nor with variables related to extra activities with voice use.

The findings highlight that proposals for preventive actions and promotion of vocal health are necessary with this population, even in the undergraduate program period, which aim to reduce the risks of developing vocal problems in the medium and (or) long term during the exercise of teaching. Here, the importance of the vocal health theme being contemplated in the curriculum of undergraduate courses is raised. Moreover, the data reinforce the importance of maintaining a continuous training, with longitudinal and specific actions such as lectures and workshops, to benefit future teachers in the awareness of voice health, which will be their main working tool throughout the professional career.
