# Supplementary material for: Future teachers and the self-perception of vocal symptoms and knowledge about vocal health and hygiene
Source: Codas. 2023 Oct 9;35(5):e20220160. doi: 10.1590/2317-1782/20232022160en (PMC10688291; doi:10.1590/2317-1782/20232022160en)
Supplement: Translation

# Translation: Futuros professores e a autopercepção de sintomas vocais e conhecimento em saúde e higiene vocal

**DOI:** 10.1590/2317-1782/20232022160pt

**Authors:** Inaiê Caroline Brugnolo Rosa, Ana Paula Dassie-Leite, Eliane Cristina Pereira, Perla do Nascimento Martins

**Affiliations:** 1 Universidade Estadual do Centro-Oeste - UNICENTRO - Irati (PR), Brasil.

**Keywords:** Voz, Docentes, Distúrbios da Voz, Promoção da Saúde, Qualidade de Vida

## Abstract

**Objetivo:**

Analisar respostas de futuros professores quanto à autopercepção de sintomas vocais e conhecimento sobre saúde vocal e higiene vocal, relacionando-os às variáveis sociodemográficas, ocupacionais e conhecimento prévio sobre voz.

**Método:**

Estudo observacional, analítico e transversal. Participaram 264 alunos de licenciatura, foram aplicados Questionário de Saúde e Higiene Vocal (QSHV), Escala de Sintomas Vocais (ESV) e questionário com perguntas sociodemográficas, ocupacionais e vocais. Os dados foram analisados estatisticamente considerando nível de significância 5%.

**Resultados:**

QSHV de futuros professores apresentou escore médio total de 21,89, sugestivo de conhecimento insuficiente sobre saúde e higiene vocal. Resultados da ESV estão acima da nota de corte do instrumento, nos três domínios e no escore total, observou-se maior sintomatologia em estudantes do primeiro ano quando comparados aos demais estudantes, nos domínios emocional, limitação e total. Houve diferença na comparação do QSHV com o ano da graduação (p=0,001), em que alunos dos terceiros e quartos anos obtiveram pontuações maiores. Observou-se correlação positiva entre escore total do QSHV e variável idade (p=0,019).

**Conclusão:**

Futuros professores apresentam sintomatologia vocal importante, compatível aos indivíduos disfônicos, e possuem conhecimento insuficiente sobre saúde e higiene vocal. O conhecimento é ainda menor em alunos dos anos de graduação iniciais e com menor idade. Futuros professores com mais idade demonstram ter mais conhecimento sobre saúde e higiene vocal. Faz-se necessária a proposição de ações preventivas à essa população, ainda no período da graduação, visando à diminuição dos riscos ao desenvolvimento de problemas vocais em médio ou longo prazo durante a docência.

## INTRODUÇÃO

Professores são profissionais da voz de alta demanda vocal e iniciam suas atividades docentes antes mesmo do término de sua formação acadêmica, durante os cursos de licenciatura. Em suas atividades laborais, a voz é considerada um dos principais recursos de trabalho, sendo que sua qualidade é de grande importância tanto para o desempenho profissional quanto para o processo ensino-aprendizagem^(1)^.

Na comparação com os demais profissionais da voz, professores destacam-se por apresentarem elevado risco para o desenvolvimento de alterações vocais^(2)^. Mais da metade dos docentes já tiveram ou possuem alguma alteração vocal^(3)^ e os principais sintomas referidos por eles são fadiga vocal, esforço vocal, cansaço ao falar, ardor, rouquidão, garganta seca, afonia, pigarro e tosse seca^(4)^.

Estudos mostram que docentes brasileiros apresentam maior percepção de sintomas vocais^(5)^, e essa condição pode estar relacionada a fatores relacionados à alta demanda vocal, fatores ambientais e ocupacionais, predisposição individual, dificuldade de aplicar o conhecimento sobre saúde vocal nas atividades cotidianas e falta de acesso à informação durante o período de formação^(6)^.

Considerando o grupo de futuros professores, há poucos estudos na literatura sobre a voz dessa população, principalmente relacionados à percepção de sintomatologia vocal^(6)^ e ao conhecimento de saúde vocal ^(7)^. Sabe-se, no entanto, que durante o período de graduação, os futuros professores raramente recebem orientações quanto à saúde vocal e tampouco possuem atividades voltadas para essa temática^(8)^.

Estudo realizado com futuros professores refere que estudantes universitários de cursos de licenciatura em pedagogia possuem maior incidência de sintomatologia vocal quando comparados com estudantes de outros cursos de graduação^(9)^. Um outro estudo refere a existência de voz rouca em discentes do último ano de cursos de licenciatura em matemática, letras e pedagogia, especialmente entre aqueles que já realizam algum tipo de atividade docente, e aponta que menos de 30% dos alunos relataram ter hábitos de cuidado com a voz^(10)^. A literatura mostra que a partir da aplicação da Escala de Sintomas Vocais (ESV) é possível constatar escores maiores, acima das notas de corte, em sujeitos com sintomas vocais quando comparados a sujeitos sem sintomas vocais^(11)^. Assim, é possível observar que sujeitos com maiores escores demonstram maior probabilidade de virem a desenvolver disfonia, demonstrando assim a importância da prevenção e (ou) de ações de promoção de saúde junto a esse grupo.

Sobre os procedimentos de saúde e higiene vocal, a literatura aponta que professores demonstram ter conhecimento básico sobre hábitos considerados benéficos ou hábitos que podem causar prejuízo para a saúde vocal^(12)^. No caso dos futuros professores, o hábito benéfico mais relatado foi o consumo de água, classificado como o maior agente de cuidado com a voz^(13)^. Vale ressaltar que a literatura refere que futuros professores mostram conhecimento vocal insuficiente^(8,13)^, principalmente para enfrentar as adversidades relacionadas ao trabalho e a ocorrência de distúrbios vocais.

Nesse contexto, para investigar a percepção do indivíduo sobre a sua própria voz, instrumentos de autoavaliação têm sido amplamente desenvolvidos e utilizados ^(14,15)^. Entre eles, o instrumento considerado mais robusto para a autoavaliação de sintomas vocais é a ESV^(16-17)^. Essa escala avalia três domínios específicos relacionados à limitação, físico e emocional, e tem sido utilizada para investigar a sintomatologia e sua relação com possíveis distúrbios vocais em profissionais da voz, como os profissionais da educação^(18)^. No caso do conhecimento sobre questões relacionadas à saúde e higiene vocal, o Questionário de Saúde e Higiene Vocal (QSHV)^(19)^ tem sido utilizado por apresentar valores de corte que separam as pessoas de acordo com o conhecimento que elas têm sobre o assunto.

Apesar da realização de pesquisas com futuros professores, e instrumentos padronizados para avaliação, ainda se observa uma lacuna significativa para a compreensão do perfil vocal e das necessidades dessa população. As pesquisas com futuros professores em geral estão ligadas diretamente a ações com viés de prevenção de disfonia^(20, 21)^, sendo que esses estudos demonstram que disfonias são comuns nos primeiros anos de licenciatura^(20)^ e que ações sobre voz possuem efeito positivo nessa população^(20,21)^. Essa condição demonstra a importância de se realizar estudos para compreender melhor as particularidades dessa população para que ações preventivas possam ser desenvolvidas. A intenção é agregar mais informações sobre o tema, principalmente na literatura brasileira, considerando que na categoria dos futuros professores a presença ou não de sintomatologia vocal, e seus conhecimentos sobre saúde e higiene vocal, ainda são pouco estudados na área da Voz. Desta forma, faz-se necessário ter mais conhecimento acerca dessa população para que se possa pensar em ações primárias voltadas para agregar conhecimento sobre saúde e higiene vocal e, consequentemente, prevenir problemas vocais em médio e (ou) longo prazo durante o exercício da docência.

Por isso, a presente pesquisa tem como objetivo analisar as respostas de futuros professores quanto à percepção de sintomas vocais e conhecimento sobre saúde vocal e higiene vocal, relacionando-as às variáveis sociodemográficas, ocupacionais e conhecimento prévio sobre voz.

## MÉTODO

### Desenho do estudo

Trata-se de um estudo observacional, analítico e transversal.

### Aspectos éticos

A pesquisa foi aprovada pelo Comitê de Ética em Pesquisa com Seres Humanos, sob o número 3.282.389.

### Amostra

Foram convidados a participar dessa pesquisa alunos matriculados em cursos de licenciatura de uma universidade pública e todos os participantes assinaram o Termo de Consentimento Livre e Esclarecido. Datas e locais foram previamente combinados entre pesquisadora e participantes e a amostra foi composta por conveniência.

Como critérios de inclusão e exclusão, as informações foram verificadas por meio de perguntas feitas aos participantes e foram considerados: 

Inclusão: ser aluno de curso de graduação, regularmente matriculado em cursos de Licenciatura. 

Exclusão: possuir histórico de: cirurgia de cabeça e pescoço, problemas neurológicos com impacto na voz e na comunicação; diagnóstico de alterações laríngeas e/ou vocais; realizar acompanhamento fonoaudiológico para reabilitação e/ou aprimoramento vocal; ser tabagista.

### Procedimentos

Os procedimentos envolvidos na coleta de dados foram as respostas aos protocolos:

- ESV - Escala de Sintomas Vocais^(16,17)^ composta por 30 questões que avaliam os seguintes domínios ligados aos sintomas vocais e impacto na vida: limitação, que avalia restrições relacionados a voz; físico, que avalia as questões biológicas e emocional, que avalia a relação do voluntário com sua voz e o impacto emocional que ela traz. O domínio limitação possui pontuação máxima de 60 pontos, o físico de 32 pontos e o emocional de 28 pontos. A pontuação máxima da ESV é de 120 pontos, sendo que quanto maior a pontuação, maior a sintomatologia vocal. As respectivas notas de corte, que separam sujeitos vocalmente saudáveis e pessoas com risco para desenvolver uma disfonia, são 11,5 para o domínio limitação, 6,5 para o domínio físico, 1,5 para o domínio emocional, e 16,0 para o escore total da ESV^(17)^.
- QSHV - Questionário de Saúde e Higiene Vocal^(18)^: composto por 31 perguntas para mensurar o conhecimento sobre saúde e higiene vocal, cada uma delas com três opções de resposta: positivo, negativo ou neutro, considerando os itens como benéficos, neutros ou prejudiciais para a voz. O escore total é calculado por somatória simples, sendo um ponto para cada resposta correta, e o valor de corte do instrumento é 23 pontos, sendo que pontuações abaixo desse valor indicam conhecimento insuficiente e consequente risco para desenvolvimento de um distúrbio vocal^(18)^.
- Questionário sobre dados sociodemográficos: as perguntas realizadas foram pertinentes a questões sociodemográficas em geral como sexo, idade, ano de graduação, atividade ocupacional/trabalho paralelamente à universidade (sim ou não), atividade externa à universidade com grande demanda vocal (trabalho e/ou outras atividades como canto em igreja, participação de grupos de canto, teatro, entre outros), e se já havia recebido orientações prévias sobre uso da voz.

### Análise de dados

Para a análise dos dados, os resultados obtidos foram tabulados em planilha EXCEL e as análises realizadas pelo software *Statistica* for Windows, versão 10.0, StatSoft Inc. Foi realizada análise descritiva dos resultados obtidos pelos estudantes no QSHV (medidas de tendência central e de dispersão). Para verificação da normalidade das variáveis contínuas foi aplicado o teste de Shapiro-Wilk, constatando-se distribuição assimétrica em todas elas. Por isso, para comparação entre os escores dos instrumentos QSHV e/ou ESV e as variáveis sexo, trabalho, outras atividades com uso da voz e orientação para o uso da voz, foi utilizado o teste não paramétrico de Mann-Whitney. A comparação entre os escores dos referidos instrumentos e a variável ano de graduação foi realizada por meio do teste não paramétrico de ANOVA de Kruskal-Wallis (mais de dois grupos), com análise post-hoc de Tukey HSD. Para a correlação entre os escores da ESV e do QSHV foi utilizado o Teste de Spearman. Em todos os testes estatísticos foi adotado o nível de significância de 5% (p<0,05).

## RESULTADOS

Participaram do presente estudo um total de 264 alunos matriculados em seis cursos de licenciatura de uma universidade. Vale mencionar que o campus universitário envolvido no estudo tem seis cursos de licenciatura, com um total de 920 vagas totais, considerando-se todos os anos de graduação. No entanto, devido à evasão e a baixa procura potencializadas nos últimos anos, sobretudo nos cursos de licenciatura, o número de alunos é bastante aquém a este total. Portanto, acredita-se que 264 participantes seja um número expressivo diante do contexto. Contudo, faz-se importante mencionarmos como uma limitação a não realização do cálculo amostral prévio, que determinaria o número exato necessário de participantes para que os resultados pudessem ser generalizados.

O grupo foi composto por alunos do primeiro ao último ano de graduação dos seguintes cursos de licenciatura: história, geografia, letras-espanhol, letras-inglês, pedagogia, matemática e educação física. Foram coletados dados de 71 alunos do primeiro (26,9%), 96 alunos do segundo (36,4%), 48 alunos do terceiro (18,1%) e 49 alunos do quarto ano (18,6%).

O grupo foi composto por 167 mulheres, 96 homens e 1 participante não informou o sexo, com média de idade de 21,86 anos, sendo mínimo de 18 anos e máximo de 47 anos. Em relação às orientações prévias sobre uso da voz, somente 38 alunos relataram terem recebido tais orientações (14,4%).

Na Tabela 1 observa-se a análise descritiva dos resultados obtidos nos protocolos ESV e QSHV. Os resultados da ESV estão acima da nota de corte do instrumento, em cada um dos três domínios e no escore total. Em relação ao QSHV, o presente estudo mostrou valor de escore total médio de 21,89, abaixo da nota de corte do instrumento.

**Tabela 1:** Análise descritiva dos escores obtidos na Escala de Sintomas Vocais (ESV) e no Questionário de Saúde e Higiene Vocal (QSHV), considerando o grupo total de futuros professores

**Escala de Sintomas Vocais (ESV)**	**n**	**Média**	**Mediana**	**Mín.**	**Máx.**	**dP**
**Físico**	264	7,29	7,00	0,00	21,00	4,49
**Emocional**	264	3,62	1,00	0,00	26,00	5,16
**Limitação**	264	17,66	16,00	0,00	47,00	8,68
**Total**	264	28,56	25,00	0,00	76,00	15,21
**Questionário de Saúde e Higiene Vocal (QSHV)**	**n**	**Média**	**Mediana**	**Mín.**	**Máx.**	**dP**
**Total**	264	21,89	24,00	0,00	31,00	6,83

**Legenda:** n = número de participantes; Mín = mínimo; Máx = máximo; dP = desvio padrão

A Tabela 2 apresenta a comparação entre os resultados da ESV e as variáveis sexo e orientações prévias sobre uso da voz, sendo possível analisar cada domínio separadamente. Conforme dados obtidos, os achados mostram que não foram observadas diferenças estatisticamente significantes para essa análise.

**Tabela 2:** Comparação da média de pontuação da ESV entre sexos, trabalho, atividades externas à universidade com grande demanda vocal (ocupacionais ou não) e orientações prévias sobre uso da voz

ESV	Mulheres (n: 167)					Homens (n: 97)					p
	Média	Mediana	Min.	Máx.	dP	Média	Mediana	Mín.	Máx.	dP	
Físico	7,46	7	0	21	4,38	7,04	6	0	21	4,68	0,343
Emocional	3,65	1	0	26	5,44	3,58	2	0	25	4,69	0,517
Limitação	18,2	16	5	47	8,78	16,8	16	0	37	8,5	0,362
Total	29,3	26	6	76	15,55	27,41	25	1	66	14,62	0,458
ESV	Orientações prévias - Não (n: 226)					Orientações prévias - Sim (n: 38)					p
	Média	Mediana	Min.	Máx.	dP	Média	Mediana	Min.	Máx.	dP	
Físico	7,14	7	0	21	4,48	8,16	8	0	18	4,51	0,167
Emocional	3,45	1	0	26	5,06	4,66	2	0	21	5,69	0,069
Limitação	17,54	16	0	47	8,64	18,34	16,5	6	39	9,05	0,706
Total	28,12	25	1	76	15	31,16	27,5	9	72	16,36	0,34
ESV	Trabalha - Não (n: 98)					Trabalha - Sim (n: 166)					p
	Média	Mediana	Min.	Máx.	dP	Média	Mediana	Mín.	Máx.	dP	
Físico	7,24	6	0	21	4,80	7,31	7	0	21	4,31	0,905
Emocional	3,91	1	0	24	5,48	3,45	1	0	26	4,97	0,489
Limitação	17,61	16	0	41	9,03	17,68	16	1	47	8,50	0,951
Total	28,45	26,5	3	72	15,96	28,45	25	1	76	14,79	0,877
ESV	Atividades externas com grande demanda vocal - Não (n: 228)					Atividades externas com grande demanda vocal - Sim (n: 36)					p
	Média	Mediana	Min.	Máx.	dP	Média	Mediana	Min.	Máx.	dP	
Físico	7,18	7	0	21	4,43	7,94	7,5	0	18	4,85	0,346
Emocional	3,68	1	0	26	5,32	3,28	2	0	16	4,08	0,668
Limitação	17,91	16	0	47	8,94	16,03	15	6	39	6,77	0,227
Total	28,76	26,00	1,00	76,00	15,54	27,25	23,00	8,00	65,00	13,03	0,58

Teste de Mann-Whitney

**Legenda:** n = número de participantes; Mín = mínimo; Máx = máximo; dP = desvio padrão; p = probabilidade de resultado conforme nível de significância

Abaixo encontram-se os resultados da comparação entre a ESV e a variável ano de graduação. Observa-se maior sintomatologia em estudantes do primeiro ano quando comparados aos estudantes dos demais anos, nos domínios emocional, limitação e total (Tabela 3).

**Tabela 3:** Comparação entre variáveis ano de graduação e média de pontuação da Escala de Sintomas Vocais (ESV), considerando o grupo de futuros professores

**Ano de Graduação**	**ESV Físico**						**post-hoc**	**ESV Emocional**						**post-hoc**
	**Média**	**Mediana**	**Min.**	**Máx.**	**dP**	**p**		**Média**	**Mediana**	**Min.**	**Máx.**	**dP**	**p**	
Primeira (n:71)	8,06	8,00	0,00	21,00	4,87	0,224		5,49	3,00	0,00	24,00	6,34	**0,005**	1>2=3
Segunda (n:96)	7,28	7,00	0,00	17,00	4,53			2,86	1,00	0,00	21,00	4,15		
Terceira (n:48)	5,88	5,00	1,00	15,00	3,20			2,04	1,00	0,00	25,00	4,07		
Quarta (n:49)	7,57	7,00	0,00	18,00	4,71			3,94	2,00	0,00	26,00	5,30		
**Ano de Graduação**	**ESV Limitação**							**ESV Total**						
	**Média**	**Mediana**	**Min.**	**Máx.**	**dP**	**P**		**Média**	**Mediana**	**Min.**	**Máx.**	**dP**	**p**	
Primeira (n:71)	20,37	19,00	1,00	47,00	9,43	**0,000**	1>3=2=4	33,89	32,00	1,00	76,00	17,56	**0,000**	1>3=2=4
Segunda (n:96)	17,46	15,50	4,00	40,00	8,11			27,60	25,00	6,00	72,00	13,18		
Terceira (n:48)	14,08	14,50	0,00	32,00	7,35			22,00	19,50	3,00	55,00	11,76		
Quarta (n:49)	17,61	17,00	4,00	38,00	8,77			29,12	23,00	6,00	75,00	15,86		

Teste Kruskal Wallis ANOVA e Teste Tukey HSD

**Legenda:** n = número de participantes; Mín = mínimo; Máx = máximo; dP = desvio padrão

Em relação à comparação entre o Escore Total do QSHV e as variáveis analisadas, a Tabela 4 mostra resultado estatisticamente significante na comparação com a variável ano de graduação (p=0,001), em que alunos dos últimos anos obtiveram maiores pontuações. Demais variáveis não apresentaram resultados significativos.

**Tabela 4:** Comparação entre média de pontuação do Questionário de Saúde e Higiene Vocal (QSHV) e variáveis sexo, ano de graduação, orientações prévias sobre uso da voz, trabalho e atividades externas à universidade com grande demanda vocal

**Variável**	**QSHV - Escore Total**							**p**	**post-hoc**
	**Grupo**	**n**	**Média**	**Mediana**	**Mín.**	**Máx.**	**dP**		
Sexo*	Fem	167	21,57	24	0	31	7,23	0,585	
	Masc	96	22,56	24	2	31	5,97		
Ano de Graduação**	1	71	19,79	22	0	30	7,61	**0,001**	4=3>1=2
	2	96	20,97	22,5	2	30	6,88		
	3	48	24,48	25,5	8	31	5,23		
	4	49	24,2	25	10	31	5,47		
Orientação prévia sobre uso da voz***	Não	226	21,83	24	0	31	6,83	0,633	
	Sim	38	22,24	24,5	4	30	6,89		
Trabalha***	Não	98	21,28	23	0	30	7,14	0,269	
	Sim	166	22,24	24	2	31	6,63		
Atividades externas à universidade com grande demanda vocal***	Não	228	21,72	24	0	31	6,87	0,319	
	Sim	36	22,94	25	2	30	6,53		

* Teste Mann-Whitney;

** Teste Kruskal Wallis ANOVA e Teste Tukey HSD;

*** u test

**Legenda:** n = número de participantes; Mín = mínimo; Máx = máximo; dP = desvio padrão

Nos achados referentes à correlação, a Tabela 5 apresenta correlação positiva significante entre o escore total do QSHV e variável idade.

**Tabela 5:** Correlação entre valor do escore total do Questionário de Saúde e Higiene Vocal (QSHV) e as variáveis idade e escore total da Escala de Sintomas Vocais (ESV), considerando o grupo de futuros professores

**Variável**	**Idade**		**ESV - Escores totais**	
	**r**	**p**	**r**	**p**
**QSHV - Escores totais**	0,144	**0,019**	-0,092	0,138

Correlação de Spearman

## DISCUSSÃO

A voz é considerada um recurso de trabalho essencial para aproximadamente 25% da população economicamente ativa^(22)^. A literatura mostra que a presença de sintomas vocais e o desconhecimento sobre procedimentos de saúde e higiene vocal podem comprometer o desempenho dos profissionais da voz^(5-6,23)^. No caso dos professores, alterações vocais podem impactar as atividades docentes e comprometer o processo de ensino-aprendizagem^(13,23)^.

A ESV do original inglês britânico VoiSS (Voice Symptom Scale) foi desenvolvida a partir de pesquisa com mais de 800 pacientes com o objetivo de ser um instrumento robusto de autoavaliação de voz e de sintomas vocais^(16)^. A versão em português é um dos instrumentos com maior poder de discriminação entre sujeitos com alterações vocais de diferentes etiologias e vozes saudáveis, seus valores de corte validados permitem o uso para triagem de grandes populações e indivíduos com risco vocal ^(17)^. Na presente pesquisa os resultados da ESV estão acima da nota de corte do instrumento, em cada um dos três domínios e no escore total, indicando alta sintomatologia vocal e risco vocal nos futuros professores.

No presente estudo, os resultados dos protocolos ESV e QSHV mostram que futuros professores apresentam importante percepção de sintomatologia vocal e pouco conhecimento sobre procedimentos de saúde e higiene vocal. No caso de futuros profissionais da voz, uma média elevada dos escores obtidos na ESV demonstra maior possibilidade de desenvolvimento de distúrbios vocais de acordo com a demanda vocal exigida pela profissão^(24)^.

A existência de sintomatologia vocal na população estudada corrobora os achados de outros estudos, os quais relatam que professores com frequência apresentam sintomas vocais, como rouquidão, dor ao falar e falhas na voz ao final do dia^(23)^. Esses sintomas podem estar relacionados a fatores como ambientes de trabalho, organização das atividades, bem como a falta de acesso a informações sobre procedimentos de saúde vocal ou impossibilidade de aplicar os conhecimentos vocais devido à organização do trabalho ou insalubridade do ambiente^(1,6,10)^.

A presença de sintomas também pode estar relacionada ao estilo de vida desses futuros professores, pois sabe-se que estudantes universitários podem apresentar alta sintomatologia vocal associada a uso vocal intenso, tabagismo, estresse e problemas digestivos^(25)^. O estudo supracitado ressalta que aspectos relacionados aos hábitos, saúde, demanda vocal e meio externo parecem influenciar o surgimento de sintomas vocais em universitários. Esses aspectos podem favorecer a ocorrência expressiva de sintomas vocais nessa população.

Em relação aos resultados do QSHV, os achados do presente estudo mostram que futuros professores demonstram menos conhecimento sobre os procedimentos relacionados ao cuidado vocal. Outros estudos realizados com futuros professores também relatam resultados semelhantes, em que os participantes relataram presença de alta sintomatologia vocal^(9)^, poucos cuidados com a voz^(10)^ e conhecimento insuficiente sobre saúde e higiene vocal^(13)^. Essa condição sugere que futuros professores necessitam de programas de orientação de saúde vocal durante o período da graduação. Tais ações são importantes, principalmente porque essa população começa a atuar profissionalmente ainda durante a formação acadêmica, por meio dos estágios, e a voz é o seu principal recurso de trabalho. A qualidade de vida influencia no bom funcionamento do organismo, inclusive para obter melhor desempenho nas atividades ocupacionais. No caso dos professores, a qualidade de vida relacionada à voz é uma importante referência para compreender a percepção que essa população tem sobre suas reações perante as alterações na voz e sobre sua saúde vocal^(24-25)^.

Nesse contexto, vale ressaltar que existem poucas discussões acerca do efeito da voz disfônica na aprendizagem dos alunos. Entretanto, uma investigação mostrou que a voz não disfônica produziu efeito positivo em detrimento dos efeitos negativos impostos pela voz disfônica do professor^(26)^. Tem-se, aí, mais um motivo pelo qual há a necessidade de dar importância à saúde vocal do professor desde o seu processo de formação, aspecto que infelizmente ainda é pouco valorizado. É perceptível a ausência de formação em voz nos cursos de licenciatura, sendo que um estudo mostrou que coordenadores de diversos setores compreendem a importância da voz como instrumento de trabalho do professor, entretanto, destacam apenas a existência de ações isoladas da grade curricular^(8)^.

Sobre a comparação entre os anos de graduação, o presente estudo aponta maior sintomatologia em estudantes do primeiro ano quando comparados aos estudantes dos demais anos, para os domínios emocional, limitação e total da ESV, compatível com sujeitos com alterações vocais. Não foram localizados estudos que compararam futuros professores em relação aos anos de graduação de licenciatura. Considerando os achados do presente estudo, sugere-se que essa diferença pode estar relacionada às mudanças referentes ao estilo de vida dessa população devido ao ingresso à universidade, pois esses futuros profissionais sofrem uma mudança em sua rotina diária, muitas vezes alteram o padrão alimentar e começam a desenvolver atividades acadêmicas que exigem maior uso vocal e melhor desempenho das habilidades comunicativas. Tais situações podem favorecer um uso vocal com mais esforço e tensão fonatória, além de gerar um aumento da insegurança e da timidez, fatores que podem influenciar na percepção de sintomas ou de desvantagem vocal^(25,27)^. Para uma melhor compreensão dessas circunstâncias, estudos fazem-se necessários para fornecerem evidências, tanto para compreender as diferenças específicas entre os anos de graduação, quanto para avaliar a voz desses futuros professores de modo multidimensional nos diferentes anos das licenciaturas.

Sobre o conhecimento de procedimentos de saúde e higiene vocal, alunos do quarto e do terceiro anos de graduação obtiveram pontuações maiores no QSHV. Esse achado sugere que quanto maior o tempo na graduação, mais conhecimento sobre saúde e higiene vocal. No caso da instituição pesquisada, todos os anos são realizadas campanhas da voz, onde são fornecidas informações sobre saúde e higiene vocal a todos os alunos dos cursos de graduação. Tais ações parecem estar aumentando o conhecimento dos alunos dos últimos anos de graduação, já que estes participaram mais vezes destas ações.

Considerando a correlação entre o escore total do QSHV e a variável idade, os resultados evidenciam que quanto maior a idade dos participantes maior o conhecimento sobre saúde e higiene vocal. Apesar da literatura apontar que sujeitos de diferentes gerações possuem basicamente o mesmo conhecimento sobre hábitos que beneficiam e prejudicam a saúde vocal^(28)^, o presente estudo mostra que quanto mais idade o estudante de licenciatura tem, maior é seu conhecimento de vida, sendo assim, é possível que o conhecimento sobre saúde e conhecimento vocal venha de experiências e vivências pessoais.

Neste estudo, não houve correlação entre o escore total do QSHV e da ESV. Um estudo recente apontou que professores com mais conhecimento sobre saúde vocal observam maior percepção de fadiga e restrição vocal^(5)^. No entanto, o referido trabalho pesquisou apenas o sintoma de fadiga. Levanta-se aqui a hipótese de que o conhecimento sobre saúde vocal nem sempre seja operativo, ou seja, conhecer determinado hábito pode não ser suficiente para modificar a prática em relação a ele e, consequentemente, prevenir possíveis sintomas vocais.

O presente estudo contribui para o conhecimento científico da área, já que mostra que futuros professores apresentam importante sintomatologia vocal e possuem falta de conhecimento sobre saúde e higiene vocal, sendo o conhecimento menor em alunos de anos iniciais, e com menor idade. Assim sendo, estratégias para a diminuição dos riscos ao desenvolvimento de problemas vocais nestes futuros profissionais da voz devem ser incentivadas. Assim, é válido ressaltar a importância do desenvolvimento de ações de saúde vocal junto a esse grupo voltado para o conhecimento de autocuidado, contribuindo para evitar possíveis problemas vocais. Compreende-se que as vivências possuem um papel importante para saber sobre saúde e higiene vocal, sendo um fator relevante não só para o desenvolvimento de hábitos e comportamentos vocais saudáveis, mas sim para a melhora da qualidade de vida dos docentes.

Portanto, é importante discutir sobre a necessidade da aproximação da Fonoaudiologia com os cursos de licenciatura, seja por meio de projeto de extensão, palestras frequentes e disciplina em grade curricular, a fim de prevenir a existência de futuros problemas de voz e garantir a efetividade de sua atividade profissional através do uso vocal adequado.

Embora não se tenha controlado de forma explícita a variável referente a atividades docentes já praticadas pelos futuros profissionais, na universidade onde os dados pesquisados, os estágios dos cursos de licenciaturas acontecem, em geral, no terceiro e quarto ano, o que permite a inferência de que, nestes anos de graduação, eles já estão desempenhando algumas funções em sala de aula. Mesmo não tendo havido diferenças estatisticamente significantes os resultados que compararam alunos com e sem atividades ocupacionais e com e sem atividades externas à universidade com grande demanda vocal, sugere-se que a variável referente ao uso vocal em outros espaços e de outros modos seja melhor investigada em pesquisas futuras, aprofundando a análise sobre possíveis sobrecargas de uso de voz tidas pelos estudantes, que poderiam influenciar nos resultados obtidos quanto à sintomatologia vocal e quanto ao conhecimento sobre a saúde da voz.

## CONCLUSÃO

O presente estudo mostra que é possível constatar a existência de sintomatologia vocal em alunos da licenciatura, quanto aos aspectos físicos, emocionais e de limitação da Escala de Sintomas Vocais, ESV. Resultados mostram que futuros professores têm pouco conhecimento sobre saúde e higiene vocal. O conhecimento é ainda menor em alunos dos anos de graduação iniciais e com menor idade, sendo perceptível que quanto maior a idade dos participantes, maior o conhecimento sobre saúde e higiene vocal. O conhecimento sobre saúde vocal não tem relação direta com os sintomas vocais apresentados pelos estudantes nem com variáveis relacionadas às atividades extras com uso da voz.

Os achados salientam que propostas de ações preventivas e de promoção de saúde vocal são necessárias junto a essa população, ainda no período da graduação, visando à diminuição dos riscos ao desenvolvimento de problemas vocais, em médio e (ou) longo prazo durante o exercício da docência. Levanta-se, aqui, a importância de o tema saúde vocal ser contemplado na grade curricular dos cursos de licenciatura. Além disso, os dados reforçam a importância de manter uma formação continuada, com ações longitudinais e específicas como palestras e oficinas, a fim de beneficiar os futuros professores na conscientização sobre a saúde da voz, que será o principal instrumento de trabalho ao longo da carreira profissional.
